# Effect of Age on Pentacam Keratoconus Indices

**DOI:** 10.1155/2018/2016564

**Published:** 2018-04-22

**Authors:** Maged Maher Salib Roshdy, Sherine Shafik Wahba, Rania Serag Elkitkat, Amira Maurice Hakim, Ramy Riad Fikry

**Affiliations:** ^1^Ophthalmology Department, Ain Shams University, Cairo, Egypt; ^2^Al Watany Eye Hospital, Cairo, Egypt; ^3^Ophthalmology Department, Cairo University, Cairo, Egypt

## Abstract

**Purpose:**

To assess the effect of age on elevation and pachymetric Pentacam keratoconus (KC) detection indices, and the need to adjust normative values accordingly.

**Methods:**

In a retrospective study, 95 eyes of myopic normal subjects without KC were evaluated using the OCULUS Pentacam, with an age range of 17.4 to 46.8 years. Subjects were categorised into three groups according to their age: the first included those younger than 21 years (19 eyes), the second was for the age range of 21–40 years (65 eyes), and the third comprised subjects older than 40 years (11 eyes).

**Results:**

There were statistically significant differences among the three groups regarding many elevation indices: AE from BFS, PE from BFS, and PE minus AE from BFS (*P* = 0.003, 0.010, and <0.001, resp.), and pachymetric indices: PPI avg, PPI max, ART avg, ART max, and diagonal decentration of the thinnest point (*P* = <0.001, 0.024, 0.003, 0.026, and 0.026, resp.). On comparing subjects below 21 years to those above 40 years, there was a statistically significant decrease of both PE from BFS and PE minus AE (*P* = 0.005 and <0.001, resp.) and statistically significant increase in AE from BFS (*P* = 0.001).

**Conclusions:**

Age is an important determinant of elevation indices, significantly altering their normative values. The use of the more robust pachymetry, rather than elevation, indices is recommended in subjects below 21 or above 40 years of age.

## 1. Introduction

As corneal refractive surgery evolves, professional expectations increase and require continuous refinements of preoperative screening and interpretation [1]. Currently, Scheimpflug tomography devices, such as the Pentacam (OCULUS Optikgerate GmbH, Wetzlar, Germany), are the most popular techniques providing anterior and posterior corneal surface elevations, together with a detailed thickness profile [2]. Early and accurate keratoconus (KC) detection using variable indices has been widely discussed, comparing the sensitivities and specificities of various parameters [3–5]. Furthermore, new algorithms and combined indices have been introduced, aiming at earlier and more precise KC detection [6, 7].

Ageing can alter the human corneal topography, with a detected increase in aberrations [8] and an altered pattern of corneal astigmatism [9]. The possible effect of age on corneal elevation and pachymetric profiles has been sparsely discussed [10].

Moreover, the spherical refractive error effect on tomographic corneal values is an issue that deserves proper analysis. Most of the topographic screening values were initially based on a predominantly myopic population [11], rendering it obviously inaccurate to apply the normative values on the hyperopic population, as emphasized in Kim et al.'s study [1].

This study aims at assessing the effect of age on elevation and pachymetry-based KC diagnostic indices and at studying this age effect after controlling for the spherical refractive error, using the OCULUS Pentacam, and the possible need to adjust the normative values accordingly.

## 2. Materials and Methods

This is a retrospective study including 95 consecutive myopic normal corneas imaged in the time interval between June 2008 and December 2009, using the Pentacam branded as Allegro Oculyzer (WaveLight, GmbH, Erlangen, Germany) [12] with software version 1.16r12, at Al Watany Eye Hospital, Cairo, Egypt. The study adhered to the tenets of the Declaration of Helsinki and was approved by the local institutional review board.

We excluded candidates with any detected corneal pathology, previous ocular surgery, contact lens wear within the last two weeks, or narrow palpebral fissure precluding proper imaging. Moreover, the participants were followed up annually until December 2016 to confirm that no ectasia developed along the years, either in eyes that underwent laser refractive surgeries (88 eyes) or in those who were unsuitable due to inconvenient myopic refractive error values (7 eyes). Hence, we made sure that any determined changes in index values were not due to forme fruste KC. Corneal evaluation on follow-up included the evaluation of refraction stability and, in query cases, by assessing the posterior elevation values, using the same Pentacam device.

Subjective refraction and spherical equivalent (SE) calculation were performed. All eyes were scanned at least thrice by Pentacam according to the recommendations of the device manual. Each scan included 25 Scheimpflug images. Despite good repeatability, data were collected from the most reliable scan as stated by the “QS” pop-up box (i.e., the largest analysed area, valid data percent, and good alignment). The data were collected from the automatically calculated indices for a reference surface shape 8 mm in diameter, then getting the elevation values on mouse click at the thinnest point. The investigated indices included:

- Elevation-based indices:
  - Anterior elevations (AE) from BFS
  - Posterior elevations (PE) from BFS
  - PE from the best-fit toric ellipsoid (BFTE)
  - PE minus AE from BFS
- Pachymetry-based indices:
  - Apex thickness, thickness at pupil centroid (CCT), and the thinnest-point thickness (TCT)
  - Minimum, average, and maximum corneal pachymetry progression indices (PPI min, PPI avg, and PPI max, resp.)
  - Minimum, average, and maximum Ambrosio's relational thickness indices (ART min, ART avg, and ART max, resp.)
  - Thinnest-point displacement at *x*- and *y*-axes (thinnest dX and thinnest dY, resp.) and diagonal decentration index

Subjects were then categorised into three groups according to their age on the day of Pentacam evaluation: the first included those younger than 21 years (19 eyes), the second was for the age range of 21–40 years (65 eyes), and the third comprised subjects older than 40 years (11 eyes).

### 2.1. Statistical Analysis

Data were collected and verified, and the compound indices were calculated using Microsoft Excel 2010 (Redmond, Washington, USA). Statistical analyses were performed using IBM SPSS Statistics (v19; Armonk, NY, USA). The following tests were performed: calculation of the mean, standard deviation (SD), one-sample Kolmogorov-Smirnov test to test normality, independent-sample Kruskal-Wallis test for comparison of the three groups, independent-sample Mann–Whitney *U* test for comparing each pair of groups, Spearman correlation coefficient, and partial correlation coefficients controlling for SE and age one at a time. Values were considered statistically significant if the *P* value was less than 0.05.

## 3. Results

### 3.1. Demographics

The study included 95 participants, with an average age of 28.7 ± 7.8 years (ranging from 17.4 to 46.8). Forty-nine right eyes and 46 left eyes were examined. Participants' spherical equivalent had a mean of −4.6 ± 3.0 D (ranging from −0.375 to −16.625).

### 3.2. Age Grouping Effect

The SE was evenly represented in the three age groups (*P* = 0.912). There were statistically significant differences among the three groups regarding many elevation (AE from BFS, PE from BFS, and PE minus AE from BFS) and pachymetric indices (PPI avg, PPI max, ART avg, ART max, and diagonal decentration of the thinnest point). On the other hand, some indices did not show statistically significant differences as regard age grouping, including a single elevation-based index (PE from BFTE) and some pachymetric indices (apex thickness, CCT, TCT, PPI min, ART min, thinnest dX, and thinnest dY) (Table 1).

The indices showing statistical significance among the three groups were then compared between every 2 groups (Table 2).

The 2 and 3 SD limits (of the indices showing statistical significance) for each of the three age groups are shown in Table 3. For ART avg and ART max, the alarming values are those less than the mean − 2 or −3 SD, while for other indices, the alarming values are those greater than the mean + 2 or +3 SD.

### 3.3. Refraction versus Age Effect

Most of indices were found correlated with SE alone (when calculating the partial correlation controlling for age). However, only the elevation indices from BFS were found correlated with age. Furthermore, on excluding the SE effect (controlling for SE), all the elevation indices from either BFS or BFTE were correlated with age (Table 4). Age was not found correlated to any of the pachymetric indices.

## 4. Discussion

Higher expectations for corneal refractive surgeries mandate better screening strategies and data analysis to avoid inappropriately permitting or excluding candidates [2]. This necessitates continuous refinements for any parameter that can cause falsely positive or negative diagnosis. Although there is a consensus on the absence of a single index robustly detecting KC and that KC diagnosis requires multiple index interpretation, adjusting and comparing the accuracies of individual indices remain an issue that deserves proper investigation. It would be highly valuable to highlight the indices to rely upon, in which cases, and their normal range.

The human cornea, together with other parts of the eye, suffers age-related changes. Some corneal topographic age changes have been previously highlighted [8, 9]. Likewise, the corneal tomographic parameters, pachymetric and elevation indices, need proper evaluation regarding their possible changes with age [10].

Most of the Pentacam normative database was obtained from refractive surgery candidates, with an age range of 21 to 40 years. The present study evaluated many pachymetry and elevation indices not only for this age range, but also for the late teenagers below 21 and for older subjects above 40 years of age, hence including a wide age range, aiming at investigating the correlation between age and various KC detection indices.

To be sure that the observed difference is not a fallacy caused by the refraction as a covariant, we performed partial correlation analyses controlled for the SE effect. This confirmed that the observed changes in indices were caused by the age effect and not only a fallacy due to refractive variation among the recruited subjects.

Regarding elevation indices, they showed statistically significant differences among groups, except for PE from BFTE. However, after controlling for the SE effect, the latter index did not stand robust in correlation to age. Thus, our results highlight a significant correlation between age and all the studied elevation indices and hence the inaccuracy of relying upon them using usual cutoff values in evaluating refractive candidates.

On evaluating elevation indices in late teenagers below 21 years, we detected a statistically significant decrease of most of the elevation indices: PE from BFS and PE minus and a statistically significant increase in AE from BFS. In the elevation indices that showed statistically significant differences among groups, the effect of age was most highlighted on comparing subjects below 21 years to those above 40 years, where all the elevation indices were statistically significant between them. Therefore, extremes of age are the most sensitive cohorts to elevation index fallacies. Regarding the older age group, more than 40 years of age, there was a tendency for higher PE and lower AE compared with younger subjects. However, this did not reach statistical significance in all cases.

Hashemi et al. [10] found a significant correlation of age with the maximum AE within the central 6 mm zone (*P* < 0.001), but not with the maximum PE (*P* = 0.476). However, their study included a heterogeneous group of patients with KC and forme fruste KC together with healthy subjects. As the values of indices in KC and forme fruste KC are highly variable, and these conditions are a confounding factor on their own, we preferred to include normal corneas only.

As regard pachymetric indices, our results revealed significant differences among groups in many of them, while some others did not show any statistical significance (apex thickness, CCT, TCT, PPI min, ART min, thinnest dX, and thinnest dY). This declares the robustness of the mentioned indices. Furthermore, on comparing every two groups, all the pachymetric indices were statistically insignificant between late teenagers below 21 and older age > 40 years. Moreover, after controlling for the SE effect, all the pachymetric indices showed no statistical significance in correlation to age. This important finding poses a recommendation of relying on pachymetric rather than elevation parameters in prerefractive surgery assessment for candidates below the age of 21 and those above 40 years of age.

Elevation-based indices were the parameters that showed a statistically significant difference across studied age groups. Hence, our study presented the two and three SD values in patients for these indices, where values outside the 2 SD limit represent less than 5% of corneas and values outside the 3 SD represent less than 0.3% of corneas. Values above 3 SD are suggestive of a probable pathology (Table 3).

Although the primary aim of our study was not to evaluate the refractive error effect, our results revealed that after controlling for the SE effect, all the elevation indices, from either BFS or BFTE, were correlated with age. This finding reaffirmed our suggestion of relying on pachymetric indices rather than elevation indices for extremes of age.

The evaluation of KC indices in relation to the SE effect has been previously discussed. Kim et al. [1] assessed corneal elevation and pachymetry in hyperopes compared to myopes, where they concluded that when adjusted for age, the PE changes remained statistically significant between hyperopic and myopic patients, but AE changes lost significance. On the contrary, Hashemi et al. [13] found that values of both maximum AE and PE within the central 4 mm circle in myopes were significantly higher than in hyperopic eyes, in a collaborative study evaluating the various effects of refractive errors on anterior segment Pentacam parameters. However, these studies analysed the SE effect between myopic and hyperopic cohorts. To the best of our knowledge, no studies analysed the effect of both age and refraction within the myopic range.

In our study, we followed up the subjects for several years, either with or without performing laser refractive surgeries, aiming to absolutely exclude forme fruste KC, an issue that may lead to fallacies in results. However, in other studies, including Kim et al. [1], they tried to avoid undiagnosed or forme fruste KC merely by excluding subjects with a family history of KC. We believe that following up patients is more reliable.

## 5. Conclusion

We recommend the use of pachymetry-based indices, or the elevation indices with altered normative data, when assessing corneas of patients outside the usual 21–40 years range.

## Figures and Tables

**Table 1:** Mean, standard deviation, and statistical significance of different indices among the three age groups.

	<21 years		21 to 40 years		>40 years		Kruskal-Wallis test (*P* value)
	Mean	SD	Mean	SD	Mean	SD	
Age	19.5	1.2	28.7	4.5	44.6	1.8	
SE	−5.24	5.01	−4.51	2.26	−4.31	2.71	
AE from BFS	2.9	1.2	2.7	1.4	1.3	1.1	0.003^∗^
PE from BFS	1.4	3.0	3.9	4.2	5.3	3.3	0.010^∗^
PE from BFTE	3.3	2.7	4.0	3.9	5.7	5.0	0.427
PE minus AE from BFS	−1.5	2.9	1.2	3.9	4.0	3.0	<0.001^∗^
Apex thickness	541.6	35.9	547.3	30.4	559.3	36.9	0.602
CCT	541.5	35.6	547.7	30.5	560.1	37.3	0.632
TCT	540.3	35.4	545.6	31.1	558.0	36.9	0.623
PPI min	0.505	0.151	0.548	0.150	0.482	0.108	0.218
PPI avg	0.779	0.132	0.845	0.129	0.700	0.089	<0.001^∗^
PPI max	1.021	0.132	1.086	0.168	0.964	0.129	0.024^∗^
ART min	1184.9	423.9	1103.7	443.5	1236.8	400.0	0.26
ART avg	713.7	134.4	661.1	110.2	812.6	143.6	0.003^∗^
ART max	538.3	81.4	513.6	82.4	588.8	90.5	0.026^∗^
Thinnest dX	0.059	0.488	0.021	0.595	−0.028	0.413	0.877
Thinnest dY	−0.222	0.159	−0.286	0.210	−0.237	0.207	0.41
Diagonal decentration	0.522	0.175	0.645	0.244	0.465	0.190	0.026^∗^

AE from BFS: anterior elevation from the best-fit sphere, PE from BFS: posterior elevation from the best-fit sphere, PE from BFTE: posterior elevation from the best-fit toric ellipsoid, CCT: thickness at pupil centroid, TCT: the thinnest-point thickness, PPI min: minimum corneal pachymetry progression index, PPI avg: average corneal pachymetry progression index, PPI max: maximum corneal pachymetry progression index, ART min: minimum Ambrosio's relational thickness index, ART avg: average Ambrosio's relational thickness index, ART max: maximum Ambrosio's relational thickness index, thinnest dX: the thinnest-point displacement at *x*-axis, thinnest dY: the thinnest-point displacement at *y*-axis. ^∗^Values flagged as statistically significant differences.

**Table 2:** The significance (*P* value) of comparing indices between every 2 groups.

Compared groups	(<21 years) versus (21–40 years)	(<21 years) versus (>40 years)	(21–40 years) versus (>40 years)
Test	Mann–Whitney	Mann–Whitney with exact significance	Mann–Whitney
AE from BFS	0.624	0.001^∗^	0.002^∗^
PE from BFS	0.022^∗^	0.005^∗^	0.092
PE minus AE from BFS	0.008^∗^	0.001^∗^	0.004^∗^
PPI avg	0.038^∗^	0.094	0.001^∗^
PPI max	0.106	0.268	0.015^∗^
ART avg	0.080	0.112	0.002^∗^
ART max	0.194	0.145	0.012^∗^
Diagonal decentration	0.061	0.471	0.025^∗^

AE from BFS: anterior elevation from the best-fit sphere, PE from BFS: posterior elevation from the best-fit sphere, PPI avg: average corneal pachymetry progression index, PPI max: maximum corneal pachymetry progression index, ART avg: average Ambrosio's relational thickness index, ART max: maximum Ambrosio's relational thickness index. ^∗^Statistical significance (*P* < 0.05).

**Table 3:** Mean ± two and three standard deviation values of the indices having statistically significant differences among different age groups.

	<21 years		21–40 years		>40 years	
	M ± 2 SD	M ± 3 SD	M ± 2 SD	M ± 3 SD	M ± 2 SD	M ± 3 SD
AE from BFS	5.4	6.6	5.6	7.0	3.5	4.6
PE from BFS	7.5	10.5	12.4	16.6	11.9	15.1
PE minus AE from BFS	4.2	7.1	9.0	12.9	10.1	13.1
PPI avg	1.042	1.174	1.102	1.231	0.879	0.968
PPI max	1.284	1.416	1.421	1.589	1.221	1.350
ART avg	444.9	310.4	440.7	330.5	525.4	381.7
ART max	375.5	294.1	348.8	266.3	407.8	317.2
Diagonal decentration	0.871	1.046	1.133	1.377	0.846	1.037

**Table 4:** Correlation of age with other indices, partial correlation with SE after controlling for age effect, and partial correlation with age after controlling for SE effect.

	Correlation of age		Correlation of SE controlled for age		Correlation of age controlled for SE	
	Spearman's rho	*P* value	Partial correlation	*P* value	Partial correlation	*P* value
AE from BFS	−0.217	0.035	0.241	0.019	−0.294	0.004
PE from BFS	0.419	0.001	0.299	0.003	0.378	0.001
PE from BFTE	0.135	0.192	0.203	0.050	0.227	0.028
PE minus AE from BFS	0.498	0.001	0.231	0.025	0.493	0.001
Apex thickness	−0.004	0.971	−0.241	0.019	0.127	0.224
CCT	0.013	0.898	−0.245	0.017	0.138	0.185
TCT	−0.013	0.899	−0.249	0.015	0.114	0.272
PPI min	0.184	0.074	0.190	0.067	0.121	0.247
PPI avg	0.062	0.553	0.285	0.005	−0.030	0.774
PPI max	0.133	0.197	0.240	0.020	0.056	0.595
ART min	−0.190	0.065	−0.200	0.053	−0.101	0.334
ART mid	−0.070	0.499	−0.364	0.001	0.125	0.232
ART max	−0.111	0.284	−0.339	0.001	0.036	0.729
Thinnest dX	−0.039	0.707	0.002	0.982	−0.039	0.711
Thinnest dY	−0.036	0.729	0.135	0.195	0.016	0.878
Resultant decentration	−0.017	0.870	0.188	0.070	−0.118	0.258
